# Effects of light-emitting diodes on muscle fatigue and exercise tolerance in patients with COPD: study protocol for a randomized controlled trial

**DOI:** 10.1186/1745-6215-14-134

**Published:** 2013-05-10

**Authors:** Eduardo Foschini Miranda, Ernesto Cesar Pinto Leal-Junior, Paulo Henrique Marchetti, Simone Dal Corso

**Affiliations:** 1 Post Graduate Program in Rehabilitation Sciences, Nove de Julho University, São Paulo, Brazil; 2 Post Graduate Program in Biophotonics Applied to Health Sciences, Nove de Julho University, São Paulo, Brazil; 3 Methodist University of Piracicaba, Rod. Luís Ometto, KM 24, Distrito Industrial Santa Bárbara D Oeste, São Paulo, SP, CEP:13451-900, Brazil; 4 Rua Vergueiro 235/249 2 subsolo, Bairro Liberdade, São Paulo, SP, CEP:01504-001, Brazil

**Keywords:** COPD, Phototherapy, LED, Muscle fatigue

## Abstract

**Background:**

Light-emitting diodes (LED) have been used to minimize muscle fatigue in athletes and healthy subjects. Patients with chronic obstructive pulmonary disease (COPD) are susceptible to early muscle fatigue.

**Objective:**

The objective of this study is to investigate the acute effects of LED on muscle function, exercise capacity and cardiorespiratory responses during isometric and dynamic exercise in patients with COPD.

**Methods:**

This study will assess 30 patients with moderate to severe obstruction (forced expiratory volume-one second,FEV_1_ ≤70% predicted). Isometric and dynamic protocols will be conducted in two visits each, for a total of four visits a week apart. First, venous blood will be taken from the patients. The isometric protocol will start with the determination of the maximum voluntary isometric contraction (MIVC) to determine the workload (60% of MIVC) for the isometric endurance test (IET). Patients will be randomized to receive either the placebo or LED application (each point will be irradiated for 30 s and the energy received at each point will be 41.7 J). Immediately after finishing this procedure, the patients will carry out the IET until the limit of tolerance or until a 20% fall of strength is observed. After the test, another blood draw will be taken. In another visit (one week later), the same order of procedures will be performed, except with the opposite (LED or placebo). For the dynamic endurance test (DET), the same procedures described above will be followed, except with 75% of the maximal workload obtained from the incremental cycle ergometer test used instead of the IET. The electromyography will be recorded during the isometric and dynamic protocols. Differences in muscle function, exercise capacity and cardiorespiratory responses between the LED and placebo applications will be analyzed. The therapeutic effects of LED could minimize muscle fatigue in patients with COPD by increasing exercise tolerance.

**Trial registration:**

Trial registration number: NCT01448564

## Background

Chronic obstructive pulmonary disease (COPD) primarily affects the lungs, but it is a systemic disease with several extra-pulmonary manifestations, most notably peripheral muscle dysfunction [1].

Skeletal muscle dysfunction in patients with COPD is characterized by a reduction of muscle strength [2] and endurance [3]. There is also a redistribution of muscle fiber types (low proportion of oxidative fibers and high proportion of glycolytic fibers) [4,5], changes in the bioenergetics (decrease of aerobic capacity, predominance of glycolytic metabolism and rapid accumulation of lactate) [6] and reduction of the capillary density and capillary/fiber ratio [7,8]. These alterations, alone or combined, are responsible for early muscle fatigue in COPD patients.

Since peripheral muscle dysfunction is one of the most serious systemic effects of COPD, strategies for improving muscle function are a priority in scientific research. In this context, phototherapy is an electrophysical intervention to be considered to prevent or postpone muscle fatigue in COPD due its effects, such as vasodilation, recruitment of collateral circulation, increased supply of oxygen and mitochondrial ATP level in the muscle [9].

Phototherapy by low-intensity laser and light-emitting diodes (LED) is a non-thermal therapy [10] characterized by a coherent and non-coherent beam of light, respectively [11]. It has been shown that both the laser and LED therapy produce similar effects due to the absorption of photons by chromophores in their tissue specific wavelengths. Currently, the LED has been presented as an alternative to the therapies that use low-intensity laser due to its low cost and smaller size [12].

The effects of phototherapy promoted by LED are mediated by the phenomenon of biostimulation [13]. In brief words, biostimulation can be explained by the absorption of energy by the intracellular chromophores [14]. The light energy is converted into chemical energy affecting positively several cellular biochemical processes [10,14].

Skeletal muscle, a tissue with a high rate of aerobic metabolism, is highly responsive to the stimulating effects of phototherapy through infrared and red rays [15]. Among the acute effects of the LED, the prevention of muscular fatigue [16] and reduced levels of lactate [17] stand out. Additionally, improvement in exercise performance and attenuation of inflammatory biochemical markers (creatine kinase, CK) and C-reactive protein (CRP) [18] have been observed. In this context, this study will investigate whether the positive effects, previously demonstrated in healthy individuals and athletes, might occur in patients with COPD. Our hypothesis is that the therapeutic effects of LED as vasodilation, improves the collateral circulation, increasing the level of oxygen content in tissues, and increased levels of mitochondrial ATP [19] could minimize muscle fatigue, increasing exercise tolerance in patients with COPD.

The primary aim of this study is to investigate the acute effects of LED phototherapy on muscle function, exercise capacity and cardiorespiratory responses during isometric (60% of maximum voluntary contraction) and dynamic (maximal incremental cycle ergometer test) exercises in patients with COPD. The secondary goal is to assess the effects of LED phototherapy on biochemical markers (CK, CRP and lactic acid).

## Methods/Design

### Study design

**Figure 1:**
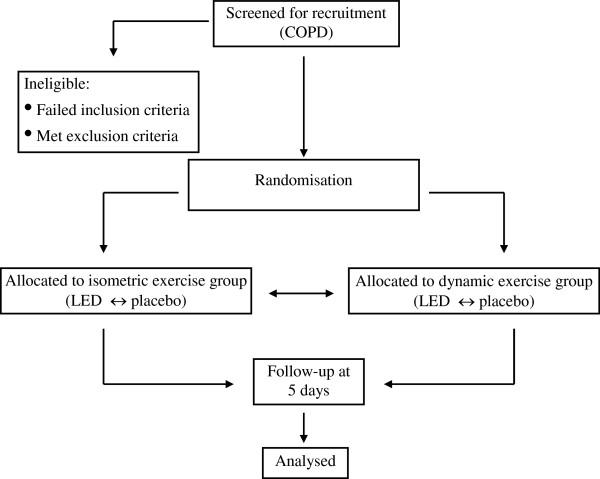
Flow of patients through the study.

This study is a crossover, double blind (patient and evaluator) and randomized trial. Randomization will be performed to determine whether the patient will receive LED or placebo (Figure 1). This study will be conducted in the Exercise Physiology Laboratory at the Nove de Julho University. This study was approved by the Ethics Committee of our institution (11/451953) and registered in Clinical Trials.gov NCT01448564 (2011-OCT-06).

The study will assess 35 patients with moderate to severe obstruction (forced expiratory volume**-**one second (FEV_1_) ≤70% predicted) and stable disease suggested by the absence of any change in medication and symptoms (increasing cough, increasing sputum volume and worsening sputum purulence) in the four weeks before the study. The exclusion criteria are oxygen-dependent patients, patients with orthopedic or neuromuscular disorders, and dark-skinned patients. Dark-skinned patients will be excluded because their greater epidermal melanin content acts as competing chromophores. This protocol was approved by the institutional ethics committee and all patients will sign a written informed consent before their participation in the study.

### Outcome measures

The primary outcome for muscle function will be the endurance time (sec) on cycling. The secondary outcomes will be the slope coefficient from the regression line between the median frequency and endurance time, fatigue and dyspnea scores (from 0 to 10), and the levels of CK (U/L), CRP (mg/dl) and lactate (mmol/L). In addition, for the dynamic protocol, the maximum workload (watts), VO_2_ peak (L), ventilation (L), and heart rate (bpm) variables will also be analyzed.

### Assessment

#### Spirometry

Spirometry will be used to classify the severity of the obstruction and it will be performed at each visit before the tests to ensure similar pulmonary function on the different days. The tests will be performed using the CPF-System (Medical Graphics Corporation®, St. Paul, MN, USA) after administering a bronchodilator (400 μg of inhaled salbutamol). The technical procedures, acceptability and reproducibility criteria will follow the recommendations of the Brazilian spirometry consensus [20]. Values of forced vital capacity and forced expiratory volume in the first secondwill be compared with those predicted for the Brazilian population. The following variables were recorded: FVC, FEV_1_, FEV_1_/FVC. The data were expressed in absolute values (liters) and in percentage of predicted (%) No, the correct sentence is like that (percentage of predict)[21]. This test will take approximately 20 minutes.

#### Skeletal muscle function assessment

The strength of the quadriceps femoris will be obtained from the dominant leg by the maximal isometric voluntary contraction (MIVC). This measurement will be made with the patients seated on a leg extension chair (Carci®, São Paulo, Brazil) at 60° of knee flexion. A non-elastic strap connecting the ankle to a load cell (EMG System EMG800C, São José dos Campos, Brazil) will be interfaced to a computer to record the MIVC. The force will be expressed in Kgf. A strap will be placed across the patient’s pelvis to minimize hip movement during the tests. The patients will perform three repetitions of MIVC of the knee extensors, each one maintained for five seconds, with a one minute rest between them. The highest value from the three reproducible contractions (<5% variability among attempts) will be considered for analysis, when the difference in strength of three contractions exceeds 5%, another measure of MIVC will be requested. The greatest value of these three contractions will be considered as the MIVC [22]. After a resting period of five minutes, the IET of the quadriceps femoris will be evaluated by the isometric endurance time at 60% of the MIVC until the limit of tolerance (Tlim). The isometric endurance test will be finished when a 20% drop of the produced force occurs. All measurements will be performed with visual feedback on the computer screen [22]. Dyspnea and leg fatigue will be evaluated before and immediately after the test by the modified Borg scale [23]. This test will take approximately 15 minutes.

#### Surface electromyography recording

Surface electromyography (sEMG) signals will be recorded (EMG System, model EMG800C, Sao José dos Campos, Brazil) during the MIVC, isometric endurance test, and the incremental cycle ergometer test. The sEMG signals will be recorded with a preamplifier (gain 1,000x) and common mode rejection of more than −85 dB. The participants’ skin will be prepared before the placement of the EMG electrodes by shaving the hair at the site of the electrode placement and cleaning the skin with alcohol [24].

Bipolar passive disposable dual Ag/AgCl snap electrodes with a 1-cm diameter for each circular conductive area and a 2-cm center-to-center spacing will be placed over the longitudinal axes of the vastuslateralis. They will be placed in the direction of the muscle fiber, taking as reference two-thirds of the distance between the anterior superior iliac spine and the lateral edge of the patella [24]. Finally, a ground electrode will be put on the left elbow.

The sampling frequency will be 2,000 Hz [25] and the criterion adopted to normalize the EMG data will be the MIVC. The digitized EMG data will first be band-pass filtered at 20 to 400 Hz using a fourth order Butterworth filter with a zero lag. For the temporal analysis, the amplitude of the EMG signals will be expressed as root mean square (RMS) (1-s moving window) and normalized by MIVC [26]. For the time-frequency analysis, the EMG data will be analyzed with a short-time Fourier transform applied to 1-s epochs [27]. The median frequency of the spectrum for each epoch will be computed, and the linear regression of the median frequencies versus time will be determined. The slope of the straight line (indicating the rate of frequency change per second) will be adopted as a second index of fatigue [28-30]. All data were analyzed using a customized program written in Matlab (Mathworks Inc., Torrance, California, EUA).

#### Cardiopulmonary exercise testing

The maximal incremental cycle ergometer test will be carried out on an electromagnetically braked cycle ergometer (Corival, LODE B.V. Medical Technology, Groningen, The Netherlands) with gas exchange and ventilatory variables being analyzed breath-by-breath (Medical Graphics Corporation (MGC), St. Paul, MN, USA). After 2 minutes at rest and more than 2 minutes with a real “zero” workload obtained by a system that moves the ergometer flywheel, the power (W) will be continuously increased in a linear “ramp” pattern (5 to 15 W min^-1^) so that the incremental exercise test duration will be between 8 and 12 minutes [29]. The following data will be obtained breath-by-breath: pulmonary oxygen uptake (VO_2_, mL min^-1^), pulmonary carbon dioxide production (VCO_2_, mL min^-1^), minute ventilation (VE, L min^-1^), tidal volume (VT, mL), respiratory rate (f, respirations per minute); ventilatory equivalent for O_2_ and CO_2_ (VE/VO_2_ and VE/VCO_2,_ respectively), and end tidal partial pressures of O_2_ and CO_2_ (PETO_2_ and PETCO_2_, mmHg). ECG tracings and heart rate (HR) will be recorded continuously and oxygen pulse (VO_2_/HR) will be calculated. Arterial oxygen saturation (SpO_2_) will be measured by pulse oximetry. Blood pressure (BP) will be measured during every two minutes of exercise. The average VO_2_ for the last 15 s of the ramp will be considered representative of the subject’s peak VO_2_. Subjects will be asked to rate dyspnea and leg fatigue at exercise cessation by using the modified Borg scale [23]. This test will take approximately 30 minutes.

#### Dynamic endurance testing

For the dynamic endurance test (DET), the same procedures described above will be followed, except with 75% of the maximal workload obtained from incremental cycle ergometer test used instead of the IET. Patients will perform this test until the limit of tolerance (Tlim).

#### Venous blood

Blood samples will be collected to measure the lactate levels, as well as the activity of CK and CRP. To measure these settings, a qualified nurse (blind to the group allocation) will perform the asepsis of ventral side of the arm. A blood sample will be collected prior to the application of the LED or placebo and immediately after ending the isometric endurance testing (Visits 1 and 2). The same procedure will be performed at Visits 3 and 4 prior to the application of the LED or placebo and after the cycle ergometer testing. The samples will be analyzed with infrared spectrophotometry via a spectrophotometer (FEMTO® Industry and Trade of Instruments, São Paulo, SP, Brazil) and specific kits for blood lactate analysis (Bioclin®, Belo Horizonte, Brazil) and CK analysis (Labtest®, Lagoa Santa, Brazil). PCR analysis will be carried out by the agglutination method using a specific analysis kit (Wiener Laboratories®, Rosario, Argentina).

#### LED

Patients will receive a single application of LED or placebo on different days (a week apart). The LED or placebo will be administered immediately before the isometric and dynamic protocols. The radiation will be held stationary with slight pressure of the LED cluster (THOR Photomedicine®, London, UK) in contact with skin at an angle of 90°. The application will be performed on two points along the ventral side of the rectus femoris [31], one point on the greater prominence of the muscle belly of the vastuslateralis and vastusmedialis muscles. For the isometric exercise protocol, the application will be performed only on the right quadriceps femoris, while for the dynamic exercise protocol, both lower limbs will receive the application on the four sites for the quadriceps femoris, two sites for the hamstring muscle belly, and one site in the center of the triceps surae [18]. Each point will be irradiated for 30 s. The energy received at each point will be 41.7 J. The irradiation settings are summarized in Table 1.

**Table 1:** Settings for LED cluster


Number of LEDs	69 (34 red LEDs and 35 infrared LEDs)
Wavelength	660 nm (red) and 850 nm (infrared)
Frequency	Continuous output
Optical output	10 mW (red) and 30 mW (infrared)
LED spot size	0.2 cm^2^ (for both), total spot sizes 13.8 cm^2^
Power density	0.05 W cm^-2^ (red) and 0.15 W cm^-2^ (infrared)
Energy	41.7 J (0.3 J from each red LED and 0.9 J from each infrared LED)
Energy density	1.5 J cm^-2^ (red) and 4.5 J cm^-2^ (infrared)
Treatment time	30 s per point, 120 s (total treatment time for isometric test), 420 s (total treatment time for dynamic test)
Number of irradiation points per muscle	4 points (isometric test) and 14 points (dynamic test)
Total energy delivered	166.8 J (isometric test), 583.8 J (dynamic test)
Application mode	Cluster held stationary to skin with slight pressure at a 90° angle

For the placebo, the same procedures will be performed but without irradiation. During the application of the LED or placebo, the patient will use protective glasses that make it impossible to see whether there is light being irradiated. This test will take approximately five minutes.

### Protocol

In the first visit, patients undergo spirometry, maximum voluntary isometric contraction (MIVC) and incremental cycle ergometer test, one hour apart. The isometric and dynamic endurance will be conducted in two visits each, totaling four visits (a week apart) with the subjects alternating between being in the test group and control group.

First, a venous blood sample will be taken from the patients. The isometric endurance test (IET) will be performed with 60% of MIVC. Then, patients will be randomized to receive either a placebo or LED application. Immediately after finishing this procedure, the patients will carry out the IET until the limit of tolerance or a fall of 20% strength is observed. After the test, another blood sample will be taken. During the next visit (one week later), the same order of procedures will be performed, except with the opposite application (LED or placebo) that was applied at Visits 2 and3.

**Figure 2:**
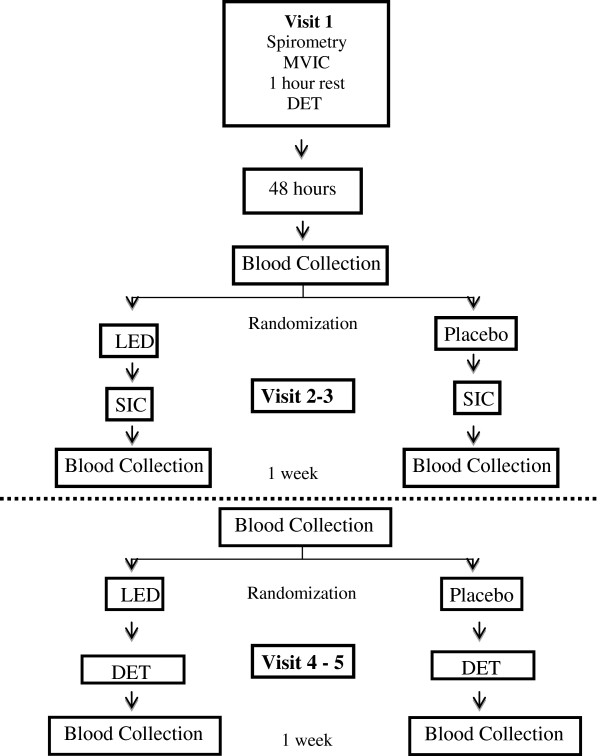
Schematic representation of the isometric and dynamic protocols.

For the dynamic endurance test (DET), the same procedures described above will be followed, except with 75% of the maximal workload obtained from incremental cycle ergometer test used instead of the IET. A summary of the protocol can be visualized in Figure 2.

### Statistical analysis

The sample size was calculated based on the primary outcome, that is, endurance time. Assuming a Type 1 error rate of 0.05 and a Type 2 error rate of 0.2, with a magnitude difference of 55 sec at the dynamic endurance testing, a standard deviation of 79 sec [32], and a hypothetical drop-out rate of 10%, 35 patients with COPD would be needed to show statistically significant differences between LED and placebo in the endurance time. It will be used as the intention to treat analysis with the baseline data being used for missing data from patients who did not complete the whole protocol [33]. An additional analysis of the results involving only those patients who complete the treatment originally allocated will also be performed (per protocol analysis).

The normal distribution of data will be verified by the Kolmogorov-Smirnov test. The parametric data will be expressed by mean and standard deviation. The non-parametric data will be expressed by median and interquartile range. Differences in the variables of muscle function, exercise capacity and cardiorespiratory responses between the LED and placebo treatments will be compared with the paired Student’s *t*-test. Changes in median frequency and root mean square will be analyzed by repeated measures analysis of variance (basal, 25%, 50%, 75% and 100% of the endurance time) [34].

The linear regression analysis of the median frequency versus endurance time will be performed to obtain the slope coefficient of the straight line. For the correlations between the electromyographic variables (median frequency and root mean square) with endurance time, peak workload at cycling, and biochemical markers (CK, CRP and lactate). Pearson’s correlation will be used for normal distributions and Spearman’s correlation will be used for non-normal distributions. We used the GraphPad Software, Inc., GraphPadInStat™(version 2.0) La Jolla, CA, USAto calculate the sample size.

## Discussion

Patients with COPD are susceptible to contractile fatigue [35]due to poor muscle endurance [35], reduced muscular capillarization [36] and early onset of lactic acidosis during exercise [37].

It is well known that fatigue and muscle injuries often occur after high intensity exercise and are accompanied by an inflammatory response. In this context, Leal Júnior*et al.*[38] showed that phototherapy can delay the onset of fatigue, probably by local mechanisms, including minimizing oxidative stress. Studies have shown that infrared laser application before high-intensity exercise can increase the removal of blood lactate and reduce muscle damage, providing a faster muscle recovery [39,40].

The reasons for the increased blood lactate removal and reduced muscular injuries after laser therapy are still uncertain. A possible explanation is that the effects of laser therapy occur by transforming light energy into chemistry and propagating their effects to the tissues and surrounding areas. The absorbed energy can act in two ways: (i) stimulating the release of autacoid substances (histamine, serotonin and bradykinin) and (ii) modifying the normal enzymatic reactions, both in the sense of excitement as inhibition [41].

Another possible mechanism behind the therapeutic effects of laser therapy is the interaction of irradiated photons with specific receptors in the mitochondria causing an increase in mitochondrial function, along with an increase in ATP (adenosine triphosphate), ribonucleic acid (RNA) and protein synthesis. This interaction leads to an increase in oxygen consumption and also in the ATP synthesis. Consequently, the phototherapy increases the cellular metabolism and, thus, decreases the inflammatory process and lactate production [11]. When absorbed by the tissues, the phototherapy can cause changes in muscle activity because of changes in the ion gradient [42], the concentration of ATP [43] and activity of Na^+^K^+^-ATPase [44].

Muscle fatigue results in the inability to maintain the metabolic process of muscle contraction. This can occur due to reduced mitochondrial energy supply to the muscle fibers. The explanation for the ability of the phototherapy to decrease muscle fatigue is that it promotes arteriolar vasodilation and improves peripheral microcirculation [45]; consequently, there is an increased supply of oxygen to muscle tissues.

The decrease in activity of the enzyme creatine kinase (CK) after phototherapy could be related to a protective effect of the phototherapy in the development of muscle ischemia. There are some indications that the phototherapy can reduce reactive oxygen species (ROS), while antioxidant levels increase [46]. Moreover, as mentioned earlier, laser therapy can stimulate the respiratory chain and the synthesis of ATP [41]. These effects, in turn, also contribute to a decrease in CK activity and the reduction of blood lactate accumulation leading to recovery from muscle fatigue [32,46-50]. Based on previous studies, phototherapy can be considered as a novel and non-invasive treatment for reduction of muscle fatigue induced by exercise in patients with COPD.

## Trial status

This study protocol is registered with ClinicalTrials.gov (NCT01448564) and this study is active, but is not yet open for participant recruitment.

## Abbreviations

ATP: Adenosine triphosphate; BP: Blood pressure; Bpm: Beats per minute; CK: Creatine kinase; CRP: C-reactive protein; COPD: Chronic obstructive pulmonary disease; DET: Dynamic endurance test; FEV1: Forced expiratory volume in 1 second; FVC: Forced vital capacity; FEV1/FVC: Ratio of FEV_1_ to FVC; HR: Heart rate; IET: Isometric endurance test; LED: Light-emitting diodes; MIVC: Maximal isometric voluntary contraction; PETCO2: End tidal partial pressure of CO_2_; PETO2: End tidal partial pressure of O_2_; RMS: Root mean square; RNA: Ribonucleic acid; ROS: Reactive oxygen species; sEMG: Surface electromyography; SIC: Submaximal isometric contraction; SpO2: Arterial oxygen saturation; Tlim: Limit of tolerance; VCO2: Pulmonary carbon dioxide production; VE: Minute ventilation; VO2: Pulmonary oxygen uptake; VT: Tidal volume

## Competing interests

The authors declare that they have no competing interests.

## Authors’ contributions

EFM and SDC designed the trial protocol and drafted the manuscript. ECPLJ and PHM contributed to the manuscript. All authors read and approved the final manuscript.
